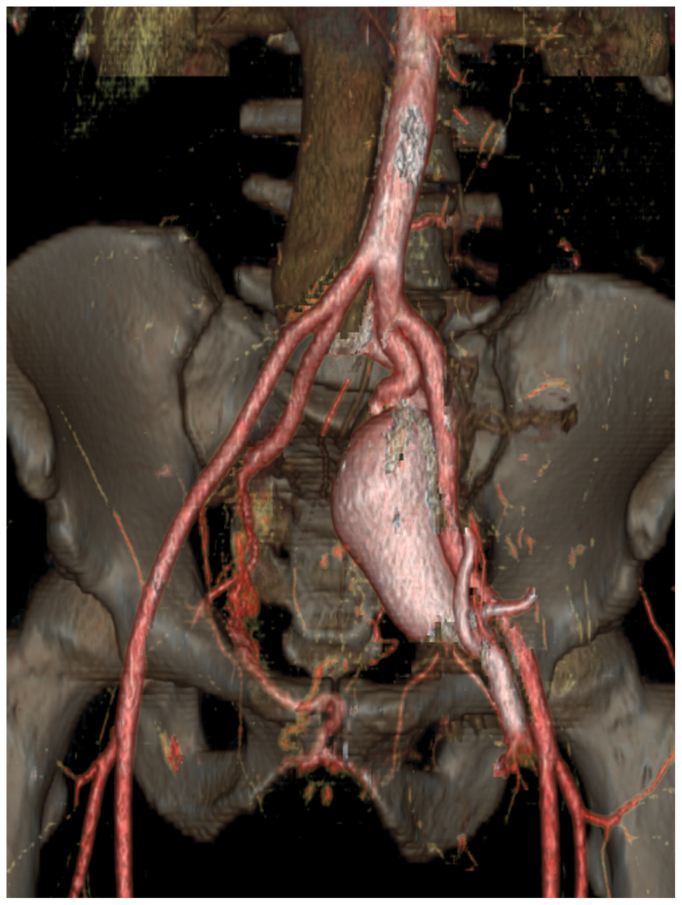# Traumatic iliac arteriovenous fistula with giant venous aneurysm following lumbar spine surgery

**DOI:** 10.1016/j.jvsv.2025.102254

**Published:** 2025-04-30

**Authors:** Aoi Ogawa, Dylan Goto, Elna Masuda

**Affiliations:** Department of Vascular Surgery, Straub Medical Center at Hawai'i Pacific Health, University of Hawai'i General Surgery Residency, Honolulu, HI

**Keywords:** Iliac vein obstruction, Venous aneurysm, Iliac arteriovenous fistula, Lumbar spine surgery

Iliac arteriovenous fistula (AVF) is a rare entity that may result from penetrating trauma including lumbar disk surgery. A 36-year-old man, who underwent lumbar disc surgery 11 and 6 years prior, presented several years later with a 4.7-cm left external iliac venous aneurysm, symptomatic iliac AVF between the left internal iliac artery and left common iliac vein, and iliac venous compression. Patient had symptoms of left leg active venous ulcers, leg swelling, and bleeding from scrotal varicose veins (*A*). He had a past surgical history of a L5-S1 discectomy owing to lumbosacral disease in 2011, as well as a repeat L5-S1 discectomy in 2016 owing to persistent symptoms. The iliac AVF was suspected to be due to penetrating trauma from the discectomy surgery given the timeline of events and no prior history of congenital symptoms.

A computed tomography angiogram showed an AVF between the left internal iliac artery and common iliac vein associated with a 4.7-cm venous external iliac vein aneurysm (*B*/Cover). He also had symptoms of May Thurner's disease owing to the underlying left iliac vein compression by the right common iliac artery. The AVF was thought to be the major contributing factor to the venous aneurysm, thus the connection between iliac artery and vein was interrupted by placing two 13 mm × 5 cm Viabahn covered stent grafts (W. L. Gore & Associates, Flagstaff, AZ) in the internal iliac artery with an overlap of >3 cm.

At 6 months postoperative, his venous ulcer completely healed and there was no further scrotal varicose vein bleeding (*C*). At 7 months postoperative, the iliac vein aneurysm decreased from 4.7 cm to 2.4 cm; however, the patient developed recurrent left leg swelling and pain secondary to the iliac vein compression; this was treated with a 16 mm × 12 cm self-expanding Abre venous stent (Medtronic, Minneapolis, MN) and resulted in symptom resolution (*D*). The patient was placed on anticoagulation for 6 months post venous stent and followed by annual duplex scan of the inferior vena cava and left lower extremity.

**Figure:**
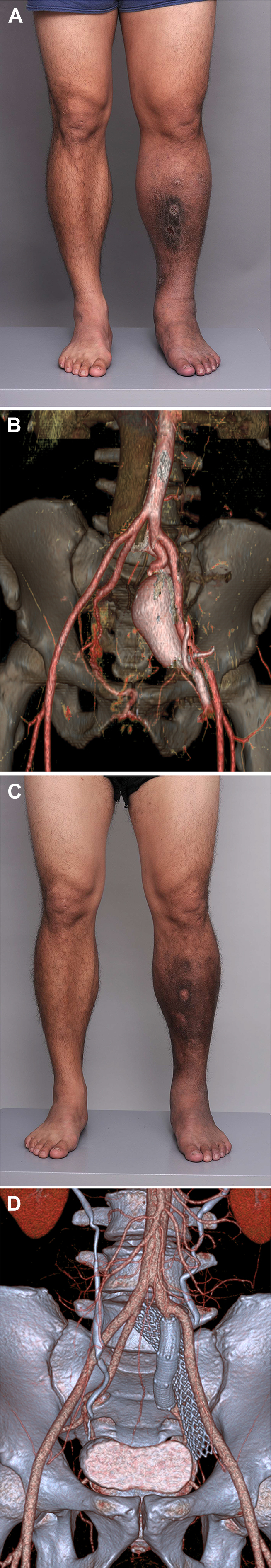

## Patient Consent

The patient has provided consent for this publication.

## Disclosures

None.

## Figures and Tables

**Cover image:**